# Supplementary material for: An open-source framework for large-scale, flexible evaluation of biomedical text mining systems
Source: J Biomed Discov Collab. 2008 Jan 29;3:1. doi: 10.1186/1747-5333-3-1 (PMC2276192; doi:10.1186/1747-5333-3-1)
Supplement: Additional file 1 — Evaluation platform source code. The additional file contains the software evaluation framework discussed in this paper. The most current release can be downloaded from SourceForge.net [1]. See the accompanying README file for instructions on installation and use. Also included in the distribution is a collection of UIMA wrappers for some commonly used BioNLP tools and annotated corpora. [file 1747-5333-3-1-S1.zip › bionlp_uima_repository-1.0.1/docs/api/src-html/edu/uchsc/ccp/uima/util/opendmap/DMAPStanfordPhraseGraph.html]

```
001    /**
002     * The OpenDMAP for Protege Project
003     * March 2006
004     */
005    package edu.uchsc.ccp.uima.util.opendmap;
006    
007    import java.io.PrintStream;
008    import java.util.ArrayList;
009    import java.util.Collection;
010    import java.util.HashMap;
011    import java.util.List;
012    
013    import org.apache.uima.cas.FSIndex;
014    import org.apache.uima.cas.FSIterator;
015    import org.apache.uima.jcas.JCas;
016    import org.apache.uima.jcas.JFSIndexRepository;
017    import org.apache.uima.jcas.cas.FSArray;
018    
019    import edu.uchsc.ccp.opendmap.DMAPPropertyFunctionException;
020    import edu.uchsc.ccp.opendmap.DMAPPropertyFunctionHandler;
021    import edu.uchsc.ccp.opendmap.DMAPToken;
022    import edu.uchsc.ccp.opendmap.Reference;
023    import edu.uchsc.ccp.uima.annotation.syntactic.CCPTokenAnnotation;
024    import edu.uchsc.ccp.uima.annotation.syntactic.CCPTypedDependency;
025    
026    /**
027     * This class packages up a Stanford Phrase Tree as a DMAP Property Function Handler.
028     * <p>
029     * The goal with this class is to make syntactic constraints that the Stanford Parser
030     * identifies available to DMAP to use as constraints between the fillers of separate
031     * slots.  For example:
032     * <pre>
033     *  {c-statement} := [subject dep:x] [action head:x] [object dep:x];
034     * </pre>
035     * This pattern is intended to mean that a c-statement can be recognized when DMAP sees
036     * a [subject] an [action] and an [object].  However, in addition, the property function
037     * constraints on [subject] and [object] says that they must be syntactically "dependent"
038     * parts of the phrase encompassing the [action] (the head of the phrase).
039     * <p>
040     * The syntactic property functions "dep" and "head" only make sense in the context of
041     * some sort of syntactic information.  This StanfordPhraseGraph class defines these two
042     * property functions and uses the information from the Stanford Parser to evaluate them.
043     * <p>
044     * In future, it is possible to add additional syntactic functions like "subj:x" and "obj:x"
045     * to use more Stanford Parser information. 
046     * <p>
047     * <i>Note</i> The main problem with this at the moment is that the Stanford Parser is 
048     * often wrong about these syntactic assignments in GeneRIFs.
049     * 
050     * @author R. James Firby
051     */
052    public class DMAPStanfordPhraseGraph implements DMAPPropertyFunctionHandler {
053    
054            /* A processed version of the Stanford Parser output highlighting dependency relationships */
055            private ParseTree tree = new ParseTree();
056            /* True if addtional debugging information should be printed */
057            private boolean debug = false;
058            
059            /* Property function constraints already seen by this handler */
060            private HashMap<String, Collection<BindingValue>> bindings = null;
061            
062            /**
063             * Private constructor used for cloning.
064             */
065            private DMAPStanfordPhraseGraph() {
066            }
067            
068            /**
069             * Create a new DMAPStanfordPhraseGraph property function handler from
070             * the annotations the Stanford Parser has left in the UIMA CAS.
071             * 
072             * @param jcas The UIMA JCas holding the Stanford Parser annotations.
073             * @param debug True if debugging information should be printed.
074             */
075            public DMAPStanfordPhraseGraph(JCas jcas, boolean debug) {
076                    this.debug = debug;
077                    // Grab an iterator over the set of CCPTokenAnnotation structures in the JCas
078                    JFSIndexRepository ir = jcas.getJFSIndexRepository();
079                    FSIndex index = ir.getAnnotationIndex(CCPTokenAnnotation.type);
080                    if (index != null) {
081                            FSIterator iterator = index.iterator();
082                            while (iterator.hasNext()) {
083                                    Object thing = iterator.next();
084                                    if (thing instanceof CCPTokenAnnotation) {
085                                            // Save dependencies between tokens to create a "phrase graph"
086                                            CCPTokenAnnotation jcasToken = (CCPTokenAnnotation) thing;
087                                            FSArray dependencies = jcasToken.getTypedDependencies();
088                                            if ((dependencies != null) && (dependencies.size() > 0)) {
089                                                    for (int i=0; i<dependencies.size(); i++) {
090                                                            thing = dependencies.get(i);
091                                                            if (thing instanceof CCPTypedDependency) {
092                                                                    CCPTypedDependency dependency = (CCPTypedDependency) thing;
093                                                                    int id = jcasToken.getTokenNumber();
094                                                                    int governor = dependency.getGovernorTokenNum();
095                                                                    int dependent = dependency.getDependentTokenNum();
096                                                                    String relation = dependency.getRelation();
097                                                                    String text = jcasToken.getCoveredText();
098                                                                    //System.out.println("Dependency: " + id + " " + text + " (" + governor + "," + dependent + ") " + relation);
099                                                                    tree.addRelation(id, governor, relation, dependent, text);
100                                                            }
101                                                    }
102                                            }
103                                    }
104                            }
105                    }
106                    tree.finalize();
107            }
108            
109            /**
110             * Check whether this property function handler wants to handle the specified function.
111             */
112            public boolean handles(String func) {
113                    if (func == null) return false;
114                    if (func.equalsIgnoreCase("dep")) return true;
115                    if (func.equalsIgnoreCase("head")) return true;
116                    return false;
117            }
118            
119            /**
120             * Evaluate a function.  That is, check to see whether the reference is consistent with
121             * this function and all functions saved away from other slots.  If all are satisfied then
122             * reference is a consistent slot filler for the slot with this property function.
123             */
124            public boolean evalFunction(String func, String arg, Reference reference) throws DMAPPropertyFunctionException {
125                    int group = getPhraseGroup(reference);
126                    String key = arg.toLowerCase();
127                    if (bindings.containsKey(key)) {
128                            Collection<BindingValue> values = bindings.get(key);
129                            return validateBindings(values, new BindingValue(func, group));
130                    } else {
131                            return true;
132                    }
133            }
134            
135            /**
136             * Record a property function constraint for later evaluation.
137             */
138            public void recordFunction(String func, String arg, Reference reference) {
139                    if (bindings == null) bindings = new HashMap<String, Collection<BindingValue>>();
140                    int group = getPhraseGroup(reference);
141                    String key = arg.toLowerCase();
142                    Collection<BindingValue> values = null;
143                    if (bindings.containsKey(key)) {
144                            values = bindings.get(key);
145                    } else {
146                            values = new ArrayList<BindingValue>(1);
147                            bindings.put(key, values);
148                    }
149                    values.add(new BindingValue(func, group));
150            }
151            
152            /**
153             * Copy this property function handler and any internal record functions.
154             */
155            public DMAPStanfordPhraseGraph clone() {
156                    DMAPStanfordPhraseGraph graph = new DMAPStanfordPhraseGraph();
157                    graph.tree = tree;
158                    graph.debug = debug;
159                    if (bindings != null) {
160                            HashMap<String, Collection<BindingValue>> newBindings = new HashMap<String, Collection<BindingValue>>();
161                            for (String key: bindings.keySet()) {
162                                    Collection<BindingValue> values = bindings.get(key);
163                                    if (values != null) {
164                                            ArrayList<BindingValue> newValues = new ArrayList<BindingValue>(values.size());
165                                            for (BindingValue value: values) newValues.add(value);
166                                            newBindings.put(key, newValues);
167                                    }
168                            }
169                            graph.bindings = newBindings;
170                    }
171                    return graph;
172            }
173            
174            /**
175             * Print a human readable version of this phrase graph.
176             * 
177             * @param stream The stream to print to.
178             */
179            public void print(PrintStream stream) {
180                    tree.print(stream);
181            }
182            
183            /**
184             * See if all the functions associated with this variable are
185             * satisfied.
186             * 
187             * @param graph The phrase graph to check in
188             * @param values The func/phrase pairs already in the bindings
189             * @param test The func/phrase pair being checked
190             * @return True if the test is consistent with all stored func/phrase pairs
191             * @throws DMAPPropertyFunctionException 
192             */
193            private boolean validateBindings(Collection<BindingValue> values, BindingValue test) throws DMAPPropertyFunctionException {
194                    if ((values == null) || (values.size() < 1)) return false;
195                    // Find the head, if there is one.
196                    int head = -1;
197                    if ((test != null) && test.function.equals("head")) {
198                            head = test.value;
199                    } else {
200                            for (BindingValue val: values) {
201                                    if (val.function.equals("head")) {
202                                            if (head != -1) {
203                                                    throw new DMAPPropertyFunctionException("Pattern found with multiple head: annotations.");
204                                            }
205                                            head = val.value;
206                                    }
207                            }
208                    }
209                    if (head == -1) return true;
210                    // Check that all dependents are children of the head
211                    boolean okay = true;
212                    if ((test != null) && test.function.equals("dep")) {
213                            okay = tree.governs(head, test.value);
214                    }
215                    if (okay) {
216                            for (BindingValue val: values) {
217                                    if (val.function.equals("dep")) {
218                                            okay = tree.governs(head, val.value);
219                                            if (!okay) break;
220                                    } else if (!val.function.equals("head")) {
221                                            throw new DMAPPropertyFunctionException("Use of unknown pattern annotation function '" + val.function + "'");
222                                    }
223                            }
224                    }
225                    return okay;
226            }
227            
228            /**
229             * Get the phrase group that this reference belongs to.  Check each token
230             * that was used in recognizing the reference to see what phrase it it in.
231             * Then find the lowest common parent that includes them all.
232             * 
233             * @param reference The reference to process.
234             * @return The phrase that this reference is part of.
235             */
236            private int getPhraseGroup(Reference reference) {
237                    // Find the phrase group for each token in the reference
238                    List<DMAPToken> tokens = reference.getTokens();
239                    if ((tokens == null) || tokens.isEmpty()) return -1;
240                    ArrayList<Integer> ids = new ArrayList<Integer>();
241                    if ((tokens.size() == 1) && (tokens.get(0).getStart() == tokens.get(0).getEnd())) {
242                            ids.add(new Integer(tokens.get(0).getStart()));
243                    } else {
244                            for (DMAPToken token: tokens) {
245                                    for (int i=token.getStart(); i<=token.getEnd(); i++) {
246                                            if (tree.isRelevantToken(i)) {
247                                                    Integer id = new Integer(i);
248                                                    if (!ids.contains(id)) ids.add(id);
249                                            }
250                                    }
251                            }
252                    }
253                    if (ids.isEmpty()) return -1;
254                    // Find the lowest common ancestor for all of these groups
255                    int realIds[] = new int[ids.size()];
256                    for (int i=0; i<ids.size(); i++) {
257                            realIds[i] = ids.get(i).intValue();
258                    }
259                    int group = tree.lowestCommonAncestor(realIds);
260                    if (group < 0) return -1;
261                    group = tree.backupAncestor(group);
262                    // Done
263                    if (debug) {
264                            System.out.println("Phrase group is " + group + " for " + reference.getReferenceString());
265                    }
266                    return group;
267            }
268    
269            /**
270             * Helper class to hold func/phrase pairs.  These represent cached property
271             * function constraints that have been saved within this handler.
272             * 
273             * @author R. James Firby
274             */
275      private class BindingValue {
276                    
277                    String function = null;
278                    int value = -1;
279                    
280                    public BindingValue(String func, int val) {
281                            function = func;
282                            value = val;
283                    }
284                    
285            }
286            
287            /**
288             * Helper class to hold the Stanford Phrase Tree
289             * <p>
290             * The phrase tree is constructed from the Stanford Parser output and highlights
291             * the dependencies between tokens.  In particular, the hierarchy represents a 
292             * set of phrases that the token belongs to.
293             * 
294             * @author R. James Firby
295             */
296            private class ParseTree {
297                    
298                    ArrayList<ParseTreeNode> roots = new ArrayList<ParseTreeNode>();
299                    ArrayList<ParseTreeNode> nodes = new ArrayList<ParseTreeNode>();
300                    
301                    /**
302                     * Add a new dependency relationship to the phrase graph.
303                     * 
304                     * @param id The identity of this relationship.  (It's phrase group number)
305                     * @param governor The identity of the governor of this phrase.
306                     * @param relation The type of relationship between the governor and the dependent.
307                     * @param dependent The identiy of the dependent node in this phrase.
308                     * @param text The text string of this phrase.
309                     */
310                    public void addRelation(int id, int governor, String relation, int dependent, String text) {
311                            ParseTreeNode newNode = new ParseTreeNode(id, governor, dependent, relation, text);
312                            boolean found = false;
313                            for (ParseTreeNode node: nodes) {
314                                    if (node.equals(newNode)) {
315                                            found = true;
316                                            break;
317                                    }
318                            }
319                            if (!found) nodes.add(newNode);
320                    }
321                    
322                    /**
323                     * Calculate all the connected subtress in the graph and cache them.
324                     */
325                    public void finalize() {
326                            for (ParseTreeNode node: nodes) {
327                                    ArrayList<ParseTreeNode> parents = findGovernors(node.id);
328                                    if (parents.isEmpty()) {
329                                            roots.add(node);
330                                    } else {
331                                            for (ParseTreeNode parent: parents) {
332                                                    parent.addChild(node);
333                                            }
334                                    }
335                            }
336                    }
337                    
338                    /**
339                     * Check whether a particular node/token governs another.
340                     * 
341                     * @param governor The identity of the possible governor.
342                     * @param dependent The identify of the possible dependent.
343                     * @return True if the dependent depends on the governor.
344                     */
345                    public boolean governs(int governor, int dependent) {
346                            for (ParseTreeNode node: nodes) {
347                                    if (node.id == governor) {
348                                            if (node.hasChild(dependent)) {
349                                                    return true;
350                                            }
351                                    }
352                            }
353                            return false;
354                    }
355                    
356                    /**
357                     * Find the lowest governor of all the dependents supplied.
358                     * 
359                     * @param children The identified of the dependent children.
360                     * @return The common ancestor (governor) of all the children.
361                     */
362                    public int lowestCommonAncestor(int children[]) {
363                            if (children.length < 1) return -1;
364                            //if (children.length == 1) return children[0];
365                            int key = children[0];
366                            int best = -1;
367                            for (ParseTreeNode node: nodes) {
368                                    if (node.id == key) {
369                                            int ancestor = lowestCommonAncestor(node, children);
370                                            if (ancestor != -1) {
371                                                    if ((best == -1) || governs(best, ancestor)) {
372                                                            best = ancestor;
373                                                    }
374                                            }
375                                    }
376                            }
377                            return best;
378                    }
379                    
380                    /**
381                     * Find the lowest common ancestor of all the children that
382                     * has the given node as a child.
383                     * 
384                     * @param node The node that must be a child.
385                     * @param children The identity of all the children.
386                     * @return The identity of the lowest common ancestor of the children looking up from node.
387                     */
388                    private int lowestCommonAncestor(ParseTreeNode node, int children[]) {
389                            ParseTreeNode lowest = null;
390                            if (governsAll(node, children)) {
391                                    lowest = node;
392                            } else {
393                                    for (ParseTreeNode ancestor: node.ancestors()) {
394                                            if (governsAll(ancestor, children)) {
395                                                    lowest = ancestor;
396                                                    break;
397                                            }
398                                    }
399                            }
400                            if (lowest == null) {
401                                    return -1;
402                            } else {
403                                    //lowest = backupAncestor(lowest);
404                                    return lowest.id;
405                            }
406                    }
407                    
408                    /**
409                     * Check whether a node governs a list of children.
410                     * 
411                     * @param node The governing node.
412                     * @param children The identities of the children.
413                     * @return True if the governing node really governs all the children.
414                     */
415                    private boolean governsAll(ParseTreeNode node, int children[]) {
416                            boolean governsAll = true;
417                            for (int i=1; i<children.length; i++) {
418                                    if (!governs(node.id, children[i])) {
419                                            governsAll = false;
420                                            break;
421                                    }
422                            }
423                            return governsAll;
424                    }
425                    
426                    /**
427                     * Backup the ancestor tree to on some types of Stanford Parser relations
428                     * to find the true governing phrase.
429                     * 
430                     * @param id The identity of the phrase to check.
431                     * @return The tru governing phrase.
432                     */
433                    public int backupAncestor(int id) {
434                            // Find the node corresponding to the given id
435                            ParseTreeNode node = null;
436                            for (ParseTreeNode n: nodes) {
437                                    if (n.id == id) {
438                                            node = n;
439                                            break;
440                                    }
441                            }
442                            if (node == null) return id;
443                            // Look for an ancestor that is the "head" of a phrase that this node belongs to.
444                            // We really want the relationships between "head" components, not individual tokens.
445                            ParseTreeNode best = node;
446                            for (ParseTreeNode parent: node.ancestors()) {
447                                    boolean okay = false;
448                                    for (ParseTreeNode child: parent.children) {
449                                            if (child.id == best.id) {
450                                                    // If the parent is the real head of a phrase that child is part of, backup.
451                                                    if (subsumes(parent, child)) {
452                                                            best = parent;
453                                                            okay = true;
454                                                    }
455                                            }
456                                    }
457                                    if (!okay) break;
458                            }
459                            return best.id;
460                    }
461                    
462                    /**
463                     * Check to see if parent is the head of a phrase that child is part of.  We are
464                     * typically looking for dependency relationships between head phrases.
465                     * 
466                     * @param parent The parent phrase node.
467                     * @param child The child phrase node.
468                     * @return True if the parent is the head of a subphrase holding child.
469                     */
470                    private boolean subsumes(ParseTreeNode parent, ParseTreeNode child) {
471                            if ((child.governor == parent.id) && child.relation.equalsIgnoreCase("nn")) {
472                                    // If the child is part of a noun phrase, (ie. "nn") then
473                                    // we really want the head noun.  So, backup.
474                                    return true;
475                            } else if ((child.governor == parent.id) && child.relation.equalsIgnoreCase("dep")) {
476                                    // If the child is a dependent subclause, then look for the root clause
477                                    return true;
478                            } else if ((child.governor == parent.id) && child.relation.equalsIgnoreCase("partmod")) {
479                                    // If the child is modified by a participle, then look for the root clause
480                                    return true;
481                            } else if ((child.governor == parent.id) && child.relation.equalsIgnoreCase("conj")) {
482                                    // If the child is part of a conjunct, then look for the root clause
483                                    return true;
484                            } else if ((child.governor == parent.id) && child.relation.equalsIgnoreCase("ccomp")) {
485                                    // If the child is part of a conjunct, then look for the root clause
486                                    return true;
487    //                      } else if ((child.governor == parent.id) && child.relation.equalsIgnoreCase("dobj")) {
488    //                              // If the child is part of a direct object, then look for the root clause
489    //                              return true;
490                            } else {
491                                    return false;
492                            }
493                    }
494                    
495                    /**
496                     * Check for Stanford Parser tokens that we should ignore.
497                     * 
498                     * @param id The id of the Stanford Parser token.
499                     * @return True if we should include this token in the phrase graph.
500                     */
501                    public boolean isRelevantToken(int id) {
502                            // Ignore "num" nodes because they are almost always labelled wrong
503                            for (int i=0; i<nodes.size(); i++) {
504                                    ParseTreeNode node = nodes.get(i);
505                                    if (node.id == id) {
506                                            if (node.relation.equalsIgnoreCase("num")) return false;
507                                    }
508                            }
509                            return true;
510                    }
511                    
512                    /**
513                     * Find all of the governors of a Stanford Parser token.
514                     * 
515                     * @param id The token id.
516                     * @return The governor nodes (akk parents from all partial subtrees).
517                     */
518                    private ArrayList<ParseTreeNode> findGovernors(int id) {
519                            ArrayList<ParseTreeNode> governors = new ArrayList<ParseTreeNode>();
520                            for (ParseTreeNode node: nodes) {
521                                    if ((node.id != id) && (node.dependent == id)) governors.add(node);
522                            }
523                            return governors;
524                    }
525                    
526                    /**
527                     * Print the parse tree in human readable form.
528                     * @param stream
529                     */
530                    public void print(PrintStream stream) {
531                            if (roots.isEmpty()) {
532                                    stream.println("No roots in parse tree.");
533                            } else {
534                                    for (ParseTreeNode root: roots) {
535                                            root.print(stream, 0);
536                                    }
537                            }
538                    }
539                    
540            }
541    
542            /**
543             * Helper class to represent a single Phrase Tree Node.
544             * 
545             * @author R. James Firby
546             */
547            private class ParseTreeNode {
548                    
549                    private int id = -1;
550                    private int governor = -1;
551                    private int dependent = -1;
552                    private String relation = null;
553                    private String text = null;
554                    
555                    /* The direct parent of this node */
556                    private ParseTreeNode parent = null;
557                    /* All of the children of this node */
558                    private ArrayList<ParseTreeNode> children = null;
559                    
560                    /* A cached list of all ancestors of this node */
561                    private ArrayList<ParseTreeNode> ancestors = null;
562                    
563                    /**
564                     * Create a new parse tree node.
565                     * 
566                     * @param id The id of this node/phrase/token.
567                     * @param governor The id of the governing token.
568                     * @param dependent The id of the dependent token.
569                     * @param relation The Stanford Parser relationship between these tokens.
570                     * @param text The text of this token.
571                     */
572                    public ParseTreeNode(int id, int governor, int dependent, String relation, String text) {
573                            this.id = id;
574                            this.governor = governor;
575                            this.dependent = dependent;
576                            this.relation = relation;
577                            this.text = text;
578                    }
579                    
580                    /**
581                     * Add a child node to this node.
582                     * 
583                     * @param node The child to add.
584                     */
585                    public void addChild(ParseTreeNode node) {
586                            ancestors = null;
587                            if (children == null) children = new ArrayList<ParseTreeNode>();
588                            children.add(node);
589                            node.parent = this;
590                    }
591                    
592                    /**
593                     * Check whether this node has a child with the given id.
594                     * 
595                     * @param childId The id of the child node.
596                     * @return True if this node has such a child.
597                     */
598                    private boolean hasChild(int childId) {
599                            if (id == childId) return true;
600                            if (children == null) return false;
601                            for (ParseTreeNode child: children) {
602                                    if (child.hasChild(childId)) {
603                                            return true;
604                                    }
605                            }
606                            return false;
607                    }
608                    
609                    /**
610                     * Compute all of the ancestors for this node and cache them away.
611                     * 
612                     * @return All ancestors for this node.
613                     */
614                    public ArrayList<ParseTreeNode> ancestors() {
615                            if (ancestors == null) {
616                                    ancestors = new ArrayList<ParseTreeNode>();
617                                    computeAncestors(ancestors, this);
618                            }
619                            return ancestors;
620                    }
621                    
622                    private void computeAncestors(ArrayList<ParseTreeNode> ancestors, ParseTreeNode node) {
623                            if (node.parent != null) {
624                                    ancestors.add(node.parent);
625                                    computeAncestors(ancestors, node.parent);
626                            }
627                    }
628                    
629                    /**
630                     * Compare two nodes to see if they represent the same information.
631                     */
632                    public boolean equals(Object thing) {
633                            if (!(thing instanceof ParseTreeNode)) return false;
634                            ParseTreeNode other = (ParseTreeNode)    thing;
635                            if (other.id != id) return false;
636                            if (other.governor != governor) return false;
637                            if (other.dependent != dependent) return false;
638                            if ((other.relation == null) && (relation == null)) return true;
639                            if ((other.relation == null) || (relation == null)) return false;
640                            return (other.relation.equalsIgnoreCase(relation));
641                    }
642                    
643                    /**
644                     * Print out this node in a human-readable format.
645                     * 
646                     * @param stream The output stream.
647                     * @param indent A prety-printing indent.
648                     */
649                    public void print(PrintStream stream, int indent) {
650                            for (int i=0; i<indent; i++) stream.print(" ");
651                            stream.print(id);
652                            stream.print(" ");
653                            stream.print(text);
654                            stream.print(" - ");
655                            stream.print(relation);
656                            stream.print(" (");
657                            stream.print(governor);
658                            stream.print(",");
659                            stream.print(dependent);
660                            stream.println(")");
661                            if (children != null) {
662                                    for (ParseTreeNode node: children) {
663                                            node.print(stream, indent + 2);
664                                    }
665                            }
666                    }
667            }
668    }
```
